# Supplementary material for: Diverging likelihood of colon and rectal cancer in Yogyakarta, Indonesia: A cross sectional study
Source: PLoS One. 2024 Mar 28;19(3):e0301191. doi: 10.1371/journal.pone.0301191 (PMC10977797; doi:10.1371/journal.pone.0301191)
Supplement: S1 Table — (PDF) [file pone.0301191.s002.pdf]

S1 Table. Parameter of data quality of colorectal cancer cases in the Yogyakarta PBCR year 2008-2019

Sub-Districts	Data Quality[1]									
	Number of cases[2]		Unknown age at diagnosis		Ill-defined sites		Unknown primary sites		Morphology verification	
	N	%	N	%	N	%	N	%	N	%
Yogyakarta PBCR all cases	16,721	100.00	5	0.03	130	0.78	344	2.06	12,822	76.68
Yogyakarta PBCR CRC cases	1,597	100.00	0	0.00	0	0.00	0	0.00	1,143	71.75
Yogyakarta PBCR CRC cases included in study	1,295	100.00	0	0.00	0	0.00	0	0.00	997	76.99
Colon cancer	402	100.00	0	0.00	0	0.00	0	0.00	328	81.59
Rectal cancer	893	100.00	0	0.00	0	0.00	0	0.00	669	74.92

Notes:

[1]No case identification was performed through death certificate collection, percentage of death certificate only (%DCO) is not calculated

[2]Data were extracted from Yogyakarta PBCR (version December 2021)
