# Supplementary material for: Diverging likelihood of colon and rectal cancer in Yogyakarta, Indonesia: A cross sectional study
Source: PLoS One. 2024 Mar 28;19(3):e0301191. doi: 10.1371/journal.pone.0301191 (PMC10977797; doi:10.1371/journal.pone.0301191)
Supplement: S2 Table — (PDF) [file pone.0301191.s003.pdf]

S2 Table. Population denominator of the Yogyakarta PBCR

Age group	Number of populations	
	Male	Female
0-4	76,350	72,320
5-9	90,432	85,101
10-14	93,321	87,688
15-19	86,314	82,517
20-24	82,662	79,912
25-29	81,203	82,570
30-34	99,022	97,553
35-39	97,600	95,558
40-44	93,364	91,417
45-49	91,913	92,635
50-54	77,534	80,402
55-59	68,202	68,083
60-64	50,399	47,707
65-69	32,127	36,479
70-74	30,608	33,196
75-79	21,822	25,447
≥80	24,191	29,110

Denominator of the Yogyakarta PBCR consisted of the number of populations of the Sleman, Kota Yogyakarta, and Bantul districts in the year 2014 as reported by the Central Bureau of Statistics of the Special Region of Yogyakarta
