# Supplementary material for: Diverging likelihood of colon and rectal cancer in Yogyakarta, Indonesia: A cross sectional study
Source: PLoS One. 2024 Mar 28;19(3):e0301191. doi: 10.1371/journal.pone.0301191 (PMC10977797; doi:10.1371/journal.pone.0301191)
Supplement: Attachment — Submitted filename: Response to Reviewer.docx [file pone.0301191.s004.docx]

**Response to the Editor and Reviewer**

Dear Editor and Reviewer,

We are thankful for the positive feedback received from the editorial team, and for the opportunity to respond to the constructive points in our submitted manuscript. Please find below our point-by-point response to the feedback outlining, where relevant, the related changes we have made. We have uploaded revised versions of the manuscript as instructed, including both a clean copy and a track changes version, and additional supplementary files made accordingly.

***Editor***

1. When submitting your revision, we need you to address these additional requirements.

Please ensure that your manuscript meets PLOS ONE's style requirements, including those for file naming. The PLOS ONE style templates can be found at

https://journals.plos.org/plosone/s/file?id=wjVg/PLOSOne_formatting_sample_main_body.pdf and

https://journals.plos.org/plosone/s/file?id=ba62/PLOSOne_formatting_sample_title_authors_affiliations.pdf

**Response**

Thank you. We have confirmed that all style formatting and file naming adheres to the PLOS ONE templates as provided in the abovementioned pages.

2. Did you know that depositing data in a repository is associated with up to a 25% citation advantage (https://doi.org/10.1371/journal.pone.0230416)? If you’ve not already done so, consider depositing your raw data in a repository to ensure your work is read, appreciated and cited by the largest possible audience. You’ll also earn an Accessible Data icon on your published paper if you deposit your data in any participating repository (https://plos.org/open-science/open-data/#accessible-data).

**Response**

We have included all minimal datasets used to produce the results presented in the manuscript as supporting information.

3. In the online submission form, you indicated that Data cannot be shared publicly because of restrictions imposed by the ethics committee as most of these contain patient data, albeit de-identified, and it may be possible to determine the identity of participants. Should there be a request for data, this can be sent to the corresponding author (email: susanna.hutajulu@ugm.ac.id)

All PLOS journals now require all data underlying the findings described in their manuscript to be freely available to other researchers, either a. In a public repository, b. Within the manuscript itself, or c. Uploaded as supplementary information.

This policy applies to all data except where public deposition would breach compliance with the protocol approved by your research ethics board. If your data cannot be made publicly available for ethical or legal reasons (e.g., public availability would compromise patient privacy), please explain your reasons on resubmission and your exemption request will be escalated for approval.

**Response**

We have now made necessary adjustment to the supplementary S1 File to also include minimal dataset to reproduce Table 1 and Table 2. All underlying data of the findings reported in the manuscript are now available in the supplementary information as required.

4. You have indicated that data is available from [email: mhrec_fmugm@ugm.ac.id]. Please can we ask you to provide us with a general contact email address for the data requests, so readers can request access in perpetuity. If a general email is not available, please provide a link to a website where readers can obtain access to data.

**Response**

Thank you for your comment. As stated in the original submission, all data access queries can be sent to the corresponding author (email: susanna.hutajulu@ugm.ac.id) or the institutional ethics committee (email: mhrec_fmugm@ugm.ac.id) at Universitas Gadjah Mada, Indonesia.

5. Please respond by return e-mail with an updated version of your manuscript to amend either the abstract on the online submission form or the abstract in the manuscript so that they are identical. We can make any changes on your behalf.

**Response**

Thank you. We confirm that we have made no changes to our abstract and will respond with the updated version of our manuscript through a return e-mail along with this resubmission.

6. Please review your reference list to ensure that it is complete and correct. If you have cited papers that have been retracted, please include the rationale for doing so in the manuscript text, or remove these references and replace them with relevant current references. Any changes to the reference list should be mentioned in the rebuttal letter that accompanies your revised manuscript. If you need to cite a retracted article, indicate the article’s retracted status in the References list and also include a citation and full reference for the retraction notice.

**Response**

Thank you for your advice. We have made sure that all references listed in the manuscript is correct and none of them have been retracted. We have added a reference as suggested by Reviewer #1 and address this in more details in the corresponding comment (Reviewer#1, comment number 3).

***Reviewer #1***

1. Please provide result of the indices of data quality of the PBCR.

**Response**

Thank you for your suggestion. We have now added S1 Table as supplementary to summarize the quality of the Yogyakarta PBCR data during the observed period.

2. Please provide a reference for the denominator.

**Response**

Thank you for your advice in elucidating the population reference used in calculating the age-standardized rate and the annual percent change of the CRC incidence in our study. Previously, we have stated in lines 187-9 under Materials and Methods that the number of populations of the Sleman, Kota Yogyakarta, and Bantul districts in 2014 was used as the denominator. Now we added the detailed number of populations in the S2 Table as supplementary.

3. Please mention the standard population with a reference used for the calculation of standardized rates.

**Response**

Thank you for your advice. We have now stated under Materials and Methods in line 191-2 that the world standard population as proposed by Segi, M (1960) was used in calculating the standardized rate. We have now also added this as reference number [31].

**Additional remarks**

We have made a correction in line 292 under Results, in which previously we refer to Fig 2 where it should be Fig 1 (middle). We confirm that this error does not affect other results presented in the manuscript.

Following our revisions, we hope the manuscript is suitable for publication in PLOS ONE.

Yours sincerely,

Susanna Hutajulu, MD, PhD
